# The association of clinical correlates, metabolic parameters, and thyroid hormones with suicide attempts in first-episode and drug-naïve patients with major depressive disorder comorbid with anxiety: a large-scale cross-sectional study

**DOI:** 10.1038/s41398-021-01234-9

**Published:** 2021-02-04

**Authors:** Yongjie Zhou, Wenchao Ren, Qianqian Sun, Katherine M. Yu, Xiaoe Lang, Zezhi Li, Xiang Yang Zhang

**Affiliations:** 1 Shenzhen Kangning Hospital, Shenzhen, Guangdong China; 2 Qingdao Mental Health Center, Qingdao University, Qingdao, China; 3 Department of Psychiatry and Behavioral Sciences, The University of Texas Health Science Center at Houston, Houston, TX USA; 4 Department of Psychiatry, The First Clinical Medical College, Shanxi Medical University, Taiyuan, China; 5 Department of Neurology, Ren Ji Hospital, Shanghai Jiao Tong University School of Medicine, Shanghai, China; 6 CAS Key Laboratory of Mental Health, Institute of Psychology, Chinese Academy of Sciences, Beijing, China; 7 Department of Psychology, University of Chinese Academy of Sciences, Beijing, China

**Keywords:** Depression, Predictive markers

## Abstract

The associated factors of suicide attempts in patients with major depressive disorder (MDD) comorbid with anxiety remains unclear. To the best of our knowledge, this is the first study with a large sample size that examines the risk factors of suicide attempts in first-episode drug-naïve (FEND) MDD patients comorbid with anxiety and includes clinical correlates, metabolic parameters, and thyroid hormone levels. A total of 1718 FEDN MDD patients were enrolled. The Hamilton Depression Scale (HAMD), Hamilton Anxiety Scale (HAMA), and Positive and Negative Syndrome Scale (PANSS) were used to assess the symptoms of patients. Metabolic parameters and thyroid hormone levels were measured. The prevalence of suicide attempts in MDD patients comorbid anxiety symptoms was 24.28%, which was 9.51 times higher than that in MDD patients without anxiety symptoms (3.25%). Compared to non-attempters, MDD patients with anxiety symptoms who attempted suicide scored higher on HAMD and HAMA, and had higher systolic blood pressure, higher levels of thyroid stimulating hormone (TSH), and thyroid peroxidases antibody (TPOAb), which were also correlated with suicide attempts in MDD patients comorbid anxiety symptoms. The combination of HAMA score, HAMD score, and TSH could differentiate suicide attempters from non-suicide attempters. Further, the age of onset, illness duration, BMI, TSH, and TPOAb were associated with the times of suicide attempts in MDD patients comorbid anxiety symptoms. Our results demonstrate high prevalence of suicide attempts in MDD patients comorbid anxiety symptoms. Several clinical correlates, metabolic parameters, and thyroid hormones function contribute to the suicide attempts in MDD patients comorbid anxiety symptoms.

## Introduction

Major depressive disorder (MDD) is a severe mental illness with high economic burden worldwide. The most severe consequence of MDD is suicide, with up to 15% of recurrent MDD individuals committing suicide even after receiving treatment with medication^1,2^. Suicide attempts are self-directed destructive behaviors with intent to die and are a serious public health challenge^3^. Previous studies have shown that 16% to 33.7% of individuals with MDD have had at least one suicide attempt in their lifetime^4–7^, which is 20-fold higher than that of the general population^8^. A recent meta-analysis shows the lifetime prevalence of suicide attempt in MDD is 31%, the 12- month prevalence rate is 8%, and the 1-month prevalence rate is 24%^9^.

Previous studies have demonstrated risk factors of suicide attempts in MDD include the male sex, severity of depression, family history of mental disorder, and comorbidities, especially with anxiety disorders^10,11^. It is worth noting that anxiety symptoms frequently occur in MDD patients, even in patients who are not diagnosed with anxiety disorder. Our recent study shows the prevalence of anxiety symptoms in first-episode and drug-naïve (FEDN) MDD patients is 79.2%^12^. Suicidal behaviors are more likely occur in MDD patients with anxiety than those without anxiety^13^. For instance, a recent meta-analysis shows that patients with anxiety disorders have more suicidal ideation (SI), attempt suicide, and complete suicide more frequently than those without anxiety disorders, even after adjustment for comorbid depression^10^. Although anxiety and anxiety symptoms are hypothetical risk factors of suicidal behavior, results on the association between suicide and anxiety or anxiety symptoms in major depression remain controversial. For example, Bolton et al. demonstrated that the frequency of suicide attempts in MDD patients with anxiety was 2.31-fold higher than that in patients without anxiety^14^. Sareen et al. found that the mood disorders with anxiety had a 2.44 times increased risk of suicide attempts than mood disorders alone^15^. However, some studies have shown that there is no link between anxiety or anxiety symptoms and suicide attempts, with even several reports that anxiety symptoms were protective factors for suicidal behavior in MDD patients^16^. These inconsistent results may be due to the heterogeneity of the samples, with variation between different episodes and psychopharmacological treatments.

FEDN MDD patients can provide us with a unique opportunity to investigate suicide attempts in MDD patients with anxiety symptoms, with minimum effects of confounding factors, including drug treatment, course of disease, and comorbid medical diseases. Furthermore, some evidence suggests that some metabolic dysfunctions^17,18^ and thyroid hormones are associated with suicide^19–21^. For example, Ma et al. showed that there was an association between low cholesterol level and suicidal attempts in 288 of inpatients with MDD^17^. Koponen et al. found that MDD patients with suicidal behavior had higher total cholesterol (TC), low-density lipoprotein cholesterol (LDL-C), triglyceride (TG), and blood glucose levels than patients without suicidal behavior^18^. Duval et al. demonstrated that the dysregulation of the HPT axis was correlated with a history of suicide^22^. Pompili et al. reported that there was a difference in FT3 levels between suicidal attempters and non-suicidal attempters of patients with psychiatric illnesses including MDD, bipolar disorder, and psychotic disorders^23^. Our previous study also showed that serum levels of thyroid stimulating hormone (TSH), anti-thyroglobulin (TgAb), and thyroid peroxidases antibody (TPOAb) were higher in MDD patients with suicide attempts compared to MDD patients without suicide attempts^19^. However, to our best knowledge, few studies have investigated the risk factors of suicide attempts in MDD patients with anxiety symptoms, while considering the effects of metabolic dysfunction or thyroid hormones.

Therefore, our present study aimed to investigate the prevalence and clinical correlates of suicide attempts in FEND MDD patients with anxiety symptoms, with an analysis of metabolic parameters and thyroid hormone levels.

## Participants and methods

### Participants

The present study was reviewed and approved by the Institutional Review Boards of First Hospital of Shanxi Medical University. Written informed consents were obtained from participants or their guardians. Patients were recruited from the Department of Psychiatry, First Clinical Medical College of Shanxi Medical University, during 2015–2017. Patients recruited in this study met the following inclusion criteria: (1) age from 18 to 60 years; (2) meeting the diagnosis of MDD based on the Diagnostic and Statistical Manual of Mental Disorders, Fourth Edition (DSM-IV); (3) first episode patients without prior drug treatment; (4) 17-item Hamilton Rating Scale for Depression (HAMD-17) score ≥24; (5) Han Chinese. Exclusion criteria included: (1) meeting any other major Axis I disorder; (2) with organic brain diseases, ongoing infections, immunosuppressive therapy, and other severe physical diseases; (3) with drug abuse or alcohol (or dependence) who were identified by self-reported drug use and medical records; (4) pregnant women.

### Clinical interview and assessment

Patients were interviewed by two independent psychiatrists through the Structure Clinical Interview for DSM-IV (SCID-I/P). The self-designed questionnaire included age, sex, height, weight, marital status, and education status and was used to collect socio-demographic data. HAMD-17 was used to assess depressive symptoms. The Hamilton Anxiety Rating Scale (HAMA) was used to assess anxiety symptoms. MDD patients with a HAMA score of 18 or greater were identified with a comorbidity of anxiety symptoms, and MDD patients with a HAMA score less than 18 were identified without anxiety symptoms^12^. The Positive and Negative Syndrome Scale (PANSS) was also applied to measure psychotic symptoms. These psychiatrists were trained on the assessment of PANSS, HAMD, and HAMA in a clinical study before the study began. The inter-rater correlation coefficient of HAMD, HAMA, and PANSS scores of psychiatrists were all more than 0.8.

In this study, lifetime suicide attempts were assessed through face-to-face interview. The question, “Have you ever attempted suicide in your lifetime?”, was derived from the WHO/EURO multicenter study^24^. The answer was encoded as “yes” or “no”. If the respondent answered ‘yes’ to this question, the following details were collected: the exact date of for each suicide attempt, the times, and the method of attempts.

### Physical and biochemical parameters measurements

The formula for body mass index (BMI) is weight in kilograms divided by height in meters squared. Fasting biochemical indexes were measured, including TC, TGs, high-density lipoprotein cholesterol (HDL-C), LDL-C, fasting blood glucose (FBG), TSH, free triiodothyronine (FT3), free thyroxine (FT4), TgAb, and TPOAb.

### Statistical analysis

Chi-square test and analysis of variance (ANOVA) were applied for categorical variables and continuous variables, respectively. Mann–Whitney *U* test was applied for non-normal distribution variables. Kolmogorov–Smirnov one-sample test was applied to detect the normal distribution. Bonferroni correction was adopted to adjust multiple tests. In order to examine the risk factors of suicide attempts in FEDN MDD patients with comorbid anxiety symptoms, a univariate analysis was applied between suicide attempters and non-attempters. Then the significantly different factors were included in the logistic regression (Backward: Wald). The area under the receiver operating characteristics (AUCROC) was used to determine the discriminatory capacity of significant parameters to distinguish between patients with and without suicide attempts. A concordance statistic between 0.7 and 0.8 was generally considered acceptable^25,26^. In addition, multivariate regression analysis was conducted to detect the association of the times of suicide attempts with the clinical and biochemical correlates of MDD with anxiety symptoms.

SPSS version 23.0 (IBM, Chicago, IL, USA) was used for analysis and then GraphPad Prism 6.0 was used to draw graphics. The *p*-values were set as two-tailed with the significance level *α* = 0.05.

## Results

### Prevalence of suicide attempts of MDD patients with comorbid anxiety symptoms vs. those without anxiety symptoms

The percentage of MDD patients with anxiety symptoms was 80.33% (1380/1718). Compared to MDD patients without anxiety symptoms, MDD patients with anxiety symptoms had higher HAMA and HAMD scores (all *p* < 0.0001). The prevalence of suicide attempts in MDD patients with anxiety symptoms (*n* = 335, 24.28%) was higher than that in MDD patients without anxiety symptoms (*n* = 11, 3.25%) (*χ*^2^ = 74.59, *p* < 0.001, OR = 10.99, 95%CI: 9.37–12.88). After controlling for HAMA and HAMD scores, the suicide attempt rate of MDD patients with anxiety symptoms increased by 9.51 times compared with MDD patients without anxiety symptoms (*B* = 2.25, Wald statistic = 51.69, *p* < 0.0001, OR = 9.51, 95%CI = 5.15–17.58).

### Clinical characteristics and biochemical parameters of suicide attempters vs. non-attempters in MDD comorbid anxiety symptoms

As shown in Table 1, univariate analysis demonstrated that there were significant differences in demographic and clinical characteristics between suicide attempters and non-attempters, including age, education, age of onset, illness duration, HAMD score, HAMA score, and PANSS positive symptom score (all *p* < 0.05). The systolic and diastolic blood pressure of the suicide attempters were higher than those of the non-suicide attempters (both *p* < 0.0001). Suicide attempters had higher levels of TC, LDL-C, FBG, TSH, TgAb, and TPOAb, but lower HDL-C compared to non-attempters (all *p* < 0.0001, all *p*_Bonferroni correction_ < 0.0001).

**Table 1:** Socio-demographics and clinical characteristics between MDD comorbid anxiety symptoms with suicide attempts and without suicide attempts.

Variable	MDD comorbid anxiety symptoms		*F/χ*^2^*/z*	*p*
	With SA (*N* = 335)	Without SA (*N* = 1045)		
Age	36.15 ± 12.33	34.57 ± 12.50	4.25	0.04
Gender			0.70	0.41
Male, *n* (%)	109 (32.5%)	366 (35%)		
Female, *n* (%)	226 (67.5%)	679 (65%)		
Education			9.55	0.02
Junior high school, *n* (%)	98 (29.3%)	234 (22.4%)		
High school, *n* (%)	135 (40.3%)	467 (44.7%)		
University degree, *n* (%)	79 (23.6%)	292 (27.9%)		
Master’s degree, *n* (%)	23 (6.9%)	52 (5.0%)		
Marital status			0.45	0.50
Single, *n* (%)	92 (27.5%)	307 (29.4%)		
Married, *n* (%)	243 (72.5%)	738 (70.6%)		
Age of onset, years	35.91 ± 12.24	34.38 ± 12.37	4.38	0.04
Illness duration, months	6.98 ± 4.93	6.11 ± 4.59	9.45	0.002
HAMD	32.36 ± 2.81	30.358 ± 2.63	131.18	<0.001
HAMA	23.83 ± 3.34	21.28 ± 2.52	209.28	<0.001
Psychotic positive score	11.62 ± 6.55	8.53 ± 3.78	107.12	<0.001
BMI, kg/m^2^	24.34 ± 2.32	24.38 ± 1.79	0.17	0.68
Systolic BP, mmHg	124.47 ± 12.05	118.70 ± 10.31	80.73	<0.001
Diastolic BP, mmHg	78.64 ± 7.70	75.64 ± 6.34	47.84	<0.001
TC, mmol/L	5.80 ± 1.11	5.23 ± 1.07	64.91	<0.001
TG, mmol/L	2.31 ± 1.01	2.16 ± 0.97	5.44	0.02^a^
HDL-C, mmol/L	1.14 ± 0.29	1.24 ± 0.29	28.99	<0.001
LDL-C, mmol/L	3.23 ± 0.91	3.01 ± 0.84	14.67	<0.001
FBG, mmol/L	5.59 ± 0.75	5.37 ± 0.63	24.44	<0.001
TSH, uIU/mL	6.78 ± 2.91	4.81 ± 2.43	143.10	<0.001
FT3, pmol/L	4.92 ± 0.73	4.91 ± 0.71	0.33	0.57
FT4, pmol/L	16.60 ± 3.14	16.72 ± 3.09	0.04	0.84
TgAb, IU/L	151.51 ± 304.32	85.30 ± 244.36	15.21	<0.001
TPOAb, IU/L	151.48 ± 249.59	57.41 ± 134.25	74.74	<0.001

*HAMD* Hamilton Rating Scale for Depression, *HAMA* Hamilton Anxiety Rating Scale, *BMI* body mass index, *BP* blood pressure, *TC* total cholesterol, *TG* triglycerides, *HDL-C* high density lipoprotein cholesterol, *LDL-C* low density lipoprotein cholesterol, *FBG* fasting blood glucose, *TSH* thyroid stimulating hormone, *FT3* free triiodothyronine, *FT4* free thyroxine, *TgAb* antithyroglobulin, *TPOAb* thyroid peroxidases antibody.

^a^Did not survive after Bonferroni correction.

### The risk factors for suicide attempts in MDD patients with anxiety symptoms

**Fig. 1:**
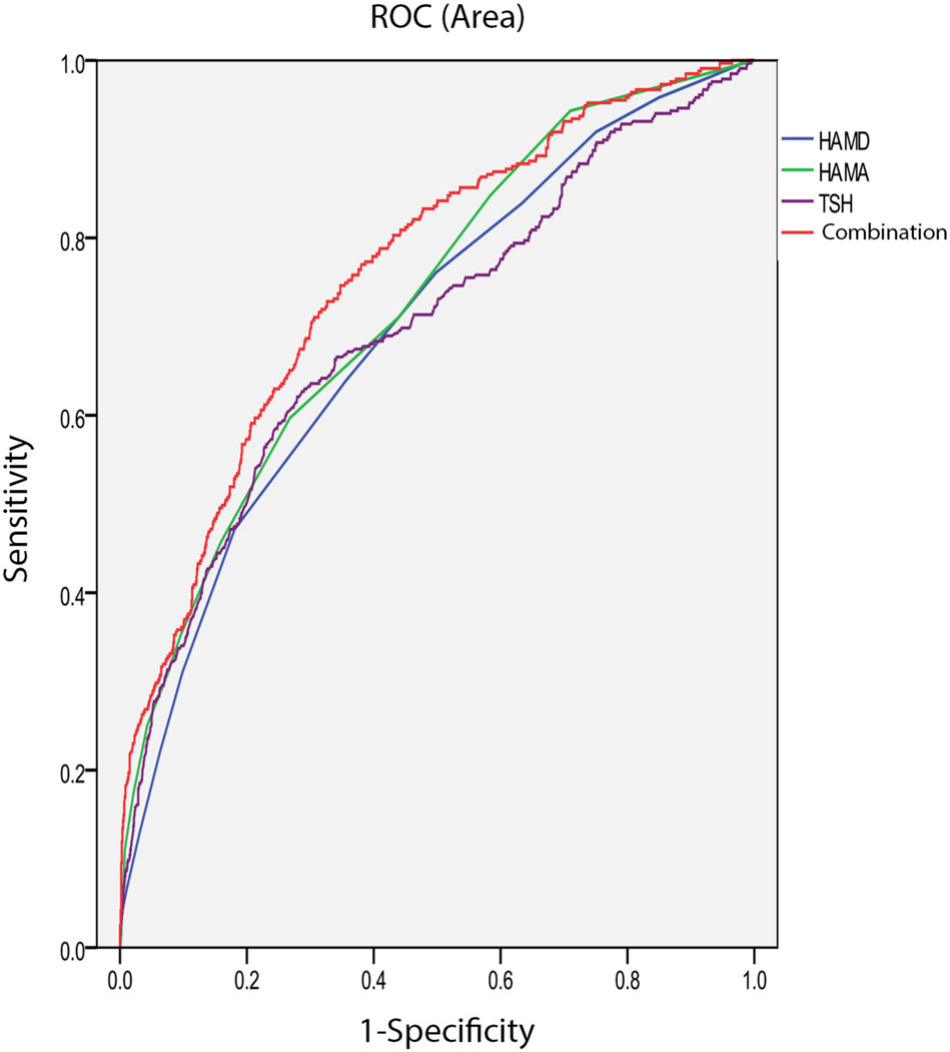
The discriminatory capacity of related factors for distinguishing between patients with and without suicide attempts in MDD comorbid with anxiety symptoms. The area under the curve of HAMD score, HAMA score, TSH, and the combination of these three factors were 0.70, 0.73, 0.70, and 0.76, respectively.

We then focused on the risk factors of suicide attempts in MDD patients with anxiety symptoms. The significantly different variables of univariate analysis were included in logistic regression (Backward: Wald) to detect the risk factors of suicide attempts in MDD patient with anxiety symptoms. As shown in Table 2, the risk factors of suicide attempts in MDD patients with anxiety symptoms were as follows: HAMA score (*B* = 0.21, *p* < 0.0001, OR = 1.23), HAMD score (*B* = 0.08, *p* = 0.02, OR = 1.08), systolic BP (*B* = 0.02, *p* = 0.005, OR = 1.02), TSH (*B* = 0.11, *p* = 0.001, OR = 1.12), TPOAb (*B* = 0.002, *p* < 0.0001, OR = 1.002). Furthermore, AUCROC showed the following values for each risk factor: HAMA was 0.73, HAMD was 0.70, TSH was 0.70, systolic BP was 0.64, and TPOAb was 0.63. Finally, when we combined the parameters with an AUC value ≥0.7, we found that the combination of HAMA score, HAMD score, and TSH had a higher AUC value of 0.76 to distinguish suicide attempters from non-attempters (*p* < 0.0001, 95%CI = 0.73–0.80) (Fig. 1).

**Table 2:** Factors associated with suicide attempts in MDD patients with anxiety symptoms.

	*B*	Wald statistic	*p*	OR	95%CI
HAMA score	0.21	51.13	<0.0001	1.23	1.16–1.30
HAMD score	0.08	5.93	0.02	1.08	1.02–1.16
Systolic BP	0.02	8.03	0.005	1.02	1.01–1.04
TSH	11.65	11.65	0.001	1.12	1.05–1.19
TPOAb	0.002	23.84	<0.0001	1.002	1.001–1.003

*HAMA* Hamilton Anxiety Rating Scale, *HAMD* Hamilton Rating Scale for Depression, *BP* blood pressure, *TSH* thyroid stimulating hormone, *TPOAb* thyroid peroxidases antibody.

### Correlation of suicide attempts times with clinical, metabolic, and thyroid hormones parameters in MDD patients with comorbid anxiety symptoms

Multivariate regression analysis showed that illness duration (*B* = 0.04, *t* = 5.97, *p* < 0.001), age of onset (*B* = 0.01, *t* = 2.45, *p* = 0.02), BMI (*B* = 0.03, *t* = 2.26, *p* = 0.02), TSH (*B* = 0.04, *t* = 2.97, *p* = 0.003), and TPOAb (*B* = 0.001, *t* = 3.18, *p* = 0.002) were independently associated with the times of suicide attempts.

## Discussion

This is the first report to investigate the suicide attempt rate and related factors in FEDN MDD patients with anxiety symptoms in a large sample size. The main results of the study were (1) the prevalence of suicide attempts in MDD patients with anxiety symptoms (24.28%) increased by 9.51 times compared with patients without anxiety symptoms (3.25%); (2) suicide attempts were associated with HAMA score, HAMD score, systolic BP, TSH, and TPOAb in MDD patients with anxiety symptoms. The combination of HAMA score, HAMD score, and TSH can discriminate between suicide attempters and non-attempters (AUC was 0.76); (3) the age of onset, illness duration, BMI, TSH, and TPOAb were associated with the times of suicide attempts in MDD patients with anxiety symptoms.

To date, only a few cross-sectional studies have reported the prevalence of suicide attempters in MDD patients with anxiety symptoms. Bartels et al. demonstrated that the prevalence of suicide ideation was higher (17.9%) in elderly MDD patients with anxiety disorders^27^. Recently, Xin et al. reported that 41 out of 910 MDD patients with anxiety symptoms had suicide attempts, showing that the prevalence of suicide attempters in MDD with anxiety symptoms was 4.51%^28^. Furthermore, evidence surrounding whether the anxiety symptoms in MDD increase the risk of suicidal behavior is inconsistent. In two previous cross-sectional studies, Bartels et al. showed that elderly MDD patients comorbid with anxiety disorders had higher rate of SI than those without anxiety disorders^27^. However, Xin et al. reported a contrary result, showing that suicide attempt rate was higher in MDD patients without anxiety symptoms than that in those with anxiety symptoms^28^. In addition, previous prospective cohort studies also yielded conflicting results. For instance, Pfeiffer et al. showed that the prevalence of suicide deaths in MDD patients with anxiety disorder was 1.25–1.27 times higher than that of MDD patients without anxiety disorder^29^. Sareen et al. demonstrated that MDD patients with anxiety disorder had a 4.15-fold increased risk of attempted suicide compared to those without anxiety disorder^15^. However, Oquendo et al. indicated that the comorbidity of anxiety did not increase the risk of suicide attempts in MDD patients^30^. Recently, Abreu et al., showed that anxiety was not associated with suicide attempts in mood disorders^31^. Furthermore, Placidi et al. showed that the comorbidity of anxiety symptoms was a protective factor for suicide in MDD patients^16^. Grunebaum et al. reported that MDD patients with attempted suicide exhibited less anxiety than those without attempted suicide^32^.

All these discrepancies may be due to the following reasons. First, socio-cultural factors may affect suicide rates and patterns, resulting in differences between Western and Eastern countries^33^. For example, in China, more women attempted and complete suicide than men^34^. In our current study, we also found that females were more likely to have suicide attempts than males in both patients with (67.5% vs. 32.5%) and without anxiety symptoms (65.0% vs. 35.0%). Second, the heterogeneity of the samples in other studies, such as different stages of disease (acute or remission) and type of depression, may affect the results. Many previous studies have documented that the number of depressive episodes^35^, and the time spent in depression^36^, impacted the incidence of suicide attempts. Compared to a complete remission phase, the relative risk of suicide attempts was 7.54 times higher during a major depressive episode, and 2.5 times higher during partial remission^36^. Several studies indicated that melancholic depression had a higher risk of suicide than depression without melancholic characters^28,37^. Third, the different antidepressants used by patients may affect the results. In addition to the difference in the efficacy of antidepressants on depression, antidepressants themselves may have different effects on suicide attempts^38^. Recent study has shown that the use of antidepressants can predict future suicide attempts^39^. Therefore, in our current study, we recruited FEDN MDD patients to exclude the effects of different disease stages and antidepressants. Fourth, the definition of anxiety symptoms was different in previous studies. For example, in STAR*D, MDD patients with HAMD-17 anxiety/somatization subscale score ≥7 were diagnosed as anxious depression^40^. Thereafter, the following studies identified the severity of anxiety symptoms in MDD patients by using HAMD-17 anxiety/somatization subscale score^41–43^. In our current study, we used a more specific assessment (HAMA scale) to measure anxiety symptoms, which was a valid and reliable indicator of the severity of anxiety in depressed patients^44^.

Multiple factors have been reported to influence suicide attempts in MDD individuals. In our present study, we found that several clinical characteristics and biochemical parameters correlated with suicide attempts in MDD patients with anxiety symptoms, including HAMA score, HAMD score, systolic BP, TSH, and TPOAb. Previous studies have consistently shown that the severity of depression and anxiety in MDD patients increased suicidal behavior. For instance, Hawton et al. conducted a meta-analysis including 28 articles and demonstrated that severe anxiety and depression were associated with an increased risk of attempted suicide in MDD patients by 1.59 times and 2.2 times, respectively^11^. Fang et al. showed that most clinical depressive symptoms were associated with the risk of suicide, although the severity of symptoms was not measured in their cross-sectional studies^45^. Recently, Hoertel et al. conducted a 3-year prospective study showing that the severity of depression independently predicted attempted suicide in MDD patients^46^. In fact, it is reasonable to conclude that the HAMD17 scale contains a suicide subscale that reflects suicide-related behaviors. In this study, we also found that the HAMA and HAMD score correlated with the times of suicide attempts, suggesting that the severity of anxiety and depression were independent risk factors of suicide attempts in FEDN MDD patients with anxiety symptoms.

As for blood pressure, some studies have investigated the association between hypertension and suicidal risk^47^. We found that systolic BP was independently and positively associated with suicide attempts in FEDN MDD patients with anxiety symptoms. A recent study has shown that the prevalence of SI was 19.6% in Chinese individuals with hypertension. Moreover, psychological distress had an impact on the relationship between SI and hypertension^47^. Psychological distress and hypertension interact to form a vicious circle, which may lead to suicide^48^. More importantly, even after adjusting for other types of physical and mental conditions, people with hypertension had an increased risk of SI^49^.

Thyroid hormones are reportedly associated with suicidal behavior in MDD; however, due to the heterogeneity of samples, the results were inconsistent^50,51^. Early studies showed that suicide attempters in MDD displayed higher TSH levels, which was positively associated with suicide attempts^52^. However, Peng et al. reported that serum TSH level was lower in MDD patients who attempted suicide^51^. In this study, we found that TSH was independently associated with suicide attempts in FEDN MDD patients with anxiety symptoms. We also showed that the TPOAb was a risk factor of suicide attempts in FEDN MDD patients with anxiety symptoms. Thyroid peroxidase (TPO) is an important enzyme for thyroid hormone production. TPO antibodies indicate an increased risk of the presence or future presence of autoimmune thyroid diseases, such as Graves’ disease and Hashimoto’s disease^53^. There were several explanations for this association as follows. First, autoimmune thyroid disease itself increases the risk of suicide in general population. For example, a recent large cohort study in the Danish population showed that the suicide mortality was increased in patients with Graves’ disease, most significantly among patients with Graves’ orbitopathy, even after adjusting for pre-existing somatic and psychiatric disease^54^. Another study of 111,565 people with Hashimoto’s disease in Danish population also showed the increased suicide mortality^55^. Second, previous studies have indicated the association of TPOAb with MDD and anxiety^56,57^, suggesting that MDD patients with higher TPOAb levels had more severe depression and anxiety symptoms, which might elevate the risk of suicide attempt. Interestingly, in this study, we also found that TSH and TPOAb were associated with the times of suicide attempts in MDD patients with anxiety symptoms. However, further studies should be conducted to investigate the mechanisms of TSH and TPOAb on the suicide risk in MDD patients.

There were some limitations in this study. First, this was a case-control design study, which prevents a causal relationship between the associated factors and suicide attempts in MDD patients with anxiety symptoms. Our results need to be confirmed by prospective cohort studies. Second, the patients included in this study were from the Han Chinese population and recruited from the outpatient clinic. Therefore, our results should be validated in other populations with different ethnic and clinical backgrounds. Third, as the severity of suicide attempts was not assessed by a structured assessment tool such as the Beck Scale for Suicidal Ideation (BSSI), future studies should examine the association between relevant risk factors and the severity of suicide behavior.

In summary, this study demonstrates that FEDN MDD patients with anxiety symptoms had a much higher incidence of suicide attempts than those MDD patients without anxiety symptoms. In FEDN MDD patients with anxiety symptoms, the severity of depression and anxiety, systolic BP, TSH, and TPOAb were risk factors for suicide attempts. Furthermore, the older age of onset, longer course of disease, greater BMI, and higher levels of TSH and TPOAb increased the times of suicide attempts in MDD patients with anxiety symptoms. However, with regard to the limitations of our cross-sectional study and lack of a structured assessment tool for suicide, our findings should be validated in future studies using a prospective cohort design.

## Data Availability

Data will be available from the corresponding author on reasonable request.
