# Correction: The association of clinical correlates, metabolic parameters, and thyroid hormones with suicide attempts in first-episode and drug-naïve patients with major depressive disorder comorbid with anxiety: a large-scale cross-sectional study

**DOI:** 10.1038/s41398-022-01935-9

**Published:** 2022-05-16

**Authors:** Yongjie Zhou, Wenchao Ren, Qianqian Sun, Katherine M. Yu, Xiaoe Lang, Zezhi Li, Xiang Yang Zhang

**Affiliations:** 1 Shenzhen Kangning Hospital, Shenzhen, Guangdong China; 2 Qingdao Mental Health Center, Qingdao University, Qingdao, China; 3 Department of Psychiatry and Behavioral Sciences, The University of Texas Health Science Center at Houston, Houston, TX USA; 4 Department of Psychiatry, The First Clinical Medical College, Shanxi Medical University, Taiyuan, China; 5 Department of Neurology, Ren Ji Hospital, Shanghai Jiao Tong University School of Medicine, Shanghai, China; 6 CAS Key Laboratory of Mental Health, Institute of Psychology, Chinese Academy of Sciences, Beijing, China; 7 Department of Psychology, University of Chinese Academy of Sciences, Beijing, China

**Keywords:** Depression, Predictive markers

Correction to: *Translational Psychiatry* 10.1038/s41398-021-01234-9, published online 04 February 2021

The original version of this article unfortunately contained a mistake. After the publication of the article the authors noticed that there is an error in an institution tag that needs to be corrected. For author Zhang Xiangyang institution tag 1 needs to be removed and the tags 6 and 7 that follow need to be retained. It should be corrected from “Zhang Xiangyang 1, 6, 7” to “Zhang Xiangyang 6, 7”. The author apologize for the error. The original article has been corrected.

